# Determinants of physical activity behaviour change in (online) interventions, and gender-specific differences: a Bayesian network model

**DOI:** 10.1186/s12966-022-01381-2

**Published:** 2022-12-19

**Authors:** Simone Catharina Maria Wilhelmina Tummers, Arjen Hommersom, Lilian Lechner, Roger Bemelmans, Catherine Adriana Wilhelmina Bolman

**Affiliations:** 1 Open University of the Netherlands, Heerlen, The Netherlands; 2 Radboud University, Nijmegen, The Netherlands; 3 Zuyd University of Applied Sciences, Heerlen, The Netherlands

**Keywords:** Physical activity, Determinants, Long- and short-term behaviour change, Differences by gender, Bayesian network, E-health intervention, Integrated dataset

## Abstract

**Background:**

Physical activity (PA) is known to be beneficial for health, but adherence to international PA guidelines is low across different subpopulations. Interventions have been designed to stimulate PA of different target groups by influencing relevant psycho-social determinants, essentially based on a combination of the Integrated Model for Change, the Theory of Planned Behaviour, its successor the Reasoned Action Approach and the self-determination theory. The current study investigates the pathways through which interventions influence PA. Further, gender differences in pathways of change are studied.

**Methods:**

An integrated dataset of five different randomised controlled trial intervention studies is analysed by estimating a Bayesian network. The data include measurements, at baseline and at 3, 6 (short-term), and 12 (long-term) months after the baseline, of important socio-cognitive determinants of PA, demographic factors, and PA outcomes. A fragment is extracted from the Bayesian network consisting of paths between the intervention variable, determinants, and short- and long-term PA outcomes. For each relationship between variables, a stability indicator and its mutual information are computed. Such a model is estimated for the full dataset, and in addition such a model is estimated based only on male and female participants’ data to investigate gender differences.

**Results:**

The general model (for the full dataset) shows complex paths, indicating that the intervention affects short-term PA via the direct determinants of intention and habit and that self-efficacy, attitude, intrinsic motivation, social influence concepts, planning and commitment have an indirect influence. The model also shows how effects are maintained in the long-term and that previous PA behaviour, intention and attitude pros are direct determinants of long-term PA. The gender-specific models show similarities as well as important differences between the structures of paths for the male- and female subpopulations. For both subpopulations, intention and habit play an important role for short-term effects and maintenance of effects in the long-term. Differences are found in the role of self-efficacy in paths of behaviour change and in the fact that attitude is relevant for males, whereas planning plays a crucial role for females. The average of these differences in subpopulation mechanisms appears to be presented in the general model.

**Conclusions:**

While previous research provided limited insight into how interventions influence PA through relevant determinants, the Bayesian network analyses show the relevance of determinants mentioned by the theoretical framework. The model clarifies the role that different determinants play, especially in interaction with each other. The Bayesian network provides new knowledge about the complex working mechanism of interventions to change PA by giving an insightful overview of influencing paths. Furthermore, by presenting subpopulation-specific networks, the difference between the influence structure of males and females is illustrated. These new insights can be used to improve interventions in order to enhance their effects. To accomplish this, we have developed a new methodology based on a Bayesian network analysis which may be applicable in various other studies.

**Supplementary Information:**

The online version contains supplementary material available at 10.1186/s12966-022-01381-2.

## Background

Physical activity (PA) is associated with important health benefits, including the prevention of chronic illnesses such as cancer, cardiovascular disease and diabetes [1–4]. It also improves cognitive functioning and mental health [5–9]. The World Health Organisation therefore recommends adults to engage in PA of moderate intensity for at least 150 min every week, spread over several days. In addition, it is recommended to execute bone- and muscle strengthening activities, supplemented with balance exercises for older adults, at least two times per week [10]. Despite these and other benefits, many people in different subpopulations do not meet the international guidelines. About 40% of European adults do not achieve the recommended levels of PA, and there is much room for improvement, particularly among relatively inactive subgroups [11–13]. In 2021, around 50% of Dutch people aged 18 and older did not meet the PA guidelines and this percentage increases with age [14]. Therefore, enhancing PA is an important strategy for improving public health.

Interventions are used to influence factors that are known to stimulate PA. If these interventions are systematically designed, and evidence- and theory-based, they have the potential to effectively increase people’s PA levels [15]. In recent years, many e-health interventions have been designed to increase PA, with demonstrated effects [16]. Some of these interventions are the subject of this paper [17–22]. The interventions included in the current paper have been designed assuming certain mechanisms of change in PA behaviour, such as change of attitude, self-efficacy, motivation, and goal setting. Effects in intervention studies, on for example PA determinants and behaviour, are measured by a collection of datasets with both intervention participants and participants in a (waitlist) control group, who have not received any intervention content during the experiment but only measurements of data. Note that the control group of each included study is comparable to the experiment group of the corresponding study and that participants of the control conditions received intervention content after the experiment phase and after the final measurements. A determinant correlates, either directly or indirectly, with PA behaviour [23]. In the latter situation, the pathway between the determinant and PA is intervened by another factor, which is called a mediator. The relation between, for example, a determinant and the PA outcome measure might vary for different groups according to levels of so-called moderators.

Studies so far have investigated the intervention effects in general on determinant and short- and long-term outcome levels as well as the statistical relevance of assumed moderators, mediators, and predictors of PA behaviour change [17–21, 24–30]. To obtain insight into the relevance and role of determinants, traditional analysis techniques have been applied in previous research, such as multilevel linear and logistic regressions, multiple mediator analyses, the conceptual mediation-moderation model, and analyses for subgroups [17–22, 24–27, 31]. Note that previous studies have mostly analysed the data of one separate study, often targeted at a specific population (e.g., older adults or [former] cancer patients). A recent systematic review and meta-analysis study focused on mediators of PA behaviour change interventions among adults [32]. In summary, they state that small, mediated effects of the intervention on PA were via beliefs about capacities, beliefs about consequences, intention, and social influences, respectively and no construct is a critical driver. For behavioural regulations, a relationship with PA was also found but did not have a significant mediation path. Although interventions were often shown effective to affect determinants and increase PA, there is still limited insight into the actual mechanisms by which the behavioural change occurs and which role key determinants play. Hence, as a result of the lack of performance of other analyses techniques than the traditional ones, little is known about a more complex structure of relations between the intervention, determinants and the PA behaviour outcome. It is essential to gain more insight into how the PA effects of these interventions were formed and if the theoretical assumptions of behavioural change mechanisms can be confirmed by empirical data. This information could be used to optimise future intervention effects, i.e. increasing PA.

It has previously been shown that there is a complex structure of interactions between interventions, determinants and PA, and that a Bayesian network model could provide a more complete and in-depth view of this structure compared to traditional analyses [33]. As Bayesian networks use a different statistical approach compared to traditional mediation analyses, it addresses a different research goal, namely to provide a comprehensive overview of relationships between variables [34]. Therefore, in this study, the modelling technique of Bayesian networks is applied [35]. Moreover, data from five different PA intervention studies are combined. This means we have much more data available, which is needed to perform Bayesian network analyses. The interventions included were previously shown to be effective and focus on different subpopulations, as outlined in Table 2, while targeting the main determinants derived from theoretical models of behavioural change. By integrating the data of multiple studies, statistical power is increased, and the combined dataset is proposed to provide a better and more general insight into relations for the overall population. Note that although our integrated datasets mainly consist of data from older adults, the subpopulations differ with respect to aspects other than age, such as physical limitations of participants. We ultimately focus on relevant parts of the Bayesian network learnt from the integrated dataset, to investigate paths of intervention influences on short- and long-term PA in more detail. The structure found is compared with the hypothesized relations of the theoretical framework behind intervention design. In this way, we aim to examine whether the data reflect the working mechanism as supposed in the theoretical framework and to gain insight into the complex structure through which the interventions influence determinants and subsequently PA.

Studies have found moderation of intervention effects on PA, for example with regard to gender and age [21, 24, 36, 37]. Therefore, we also investigate if and how the structure of paths of intervention influence change when focusing on specific subpopulations distinguished by demographic factor values. The difference between determinants of short- and long-term behaviour change, found in gender-specific subpopulation models and in the general model, is clarified in this study. Emphasis is placed on the influence of gender, since it is known that males and females have different motives and considerations to be physically active, and in previous research gender has been found to moderate intervention effects [24, 36–38]. In previous research, the role of factors associated with sedentary behaviour has been investigated similarly by estimating and visualising Bayesian networks for the complete sample and for (gender-)specific subgroups [39].

**Fig. 1:**
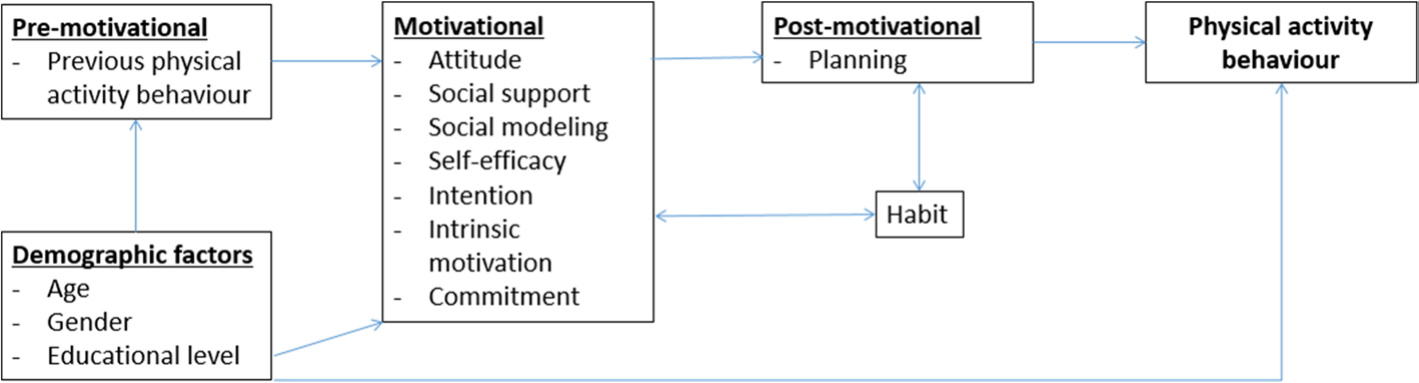
Theoretical framework of included interventions [48–50]

The intervention programmes that are the focus of this study all applied the same theoretical psychological methods to change PA and were designed following Intervention Mapping, which is a six-step protocol that facilitates a stepwise process for theory- and evidence-based development of health-promotion interventions [15]. They are designed to enhance the PA of different target (sub) populations, which mainly vary with respect to age, health status or other physical limitations, and marital status [28–30, 40, 41]. However, all interventions included are essentially based on the Integrated Model for Change (I-Change Model) [42]; the theory of planned behaviour [43]; its successor, the Reasoned Action Approach [44]; and the self-determination theory [45]. It is hypothesised that changes in PA behaviour are reached by influencing their determinants [28–30, 40, 41]. An example of these determinants is self-efficacy, the extent to which someone expects him- or herself to be capable of performing PA. Based on theoretical psychological methods, behaviour change strategies are implemented in the intervention programmes in order to influence the hypothesized important psycho-social determinants of PA behaviour [15, 46], see Table 1. The supposed role of the determinants in influencing PA is shown in the theoretical framework depicted in Fig. 1, which summarises results from previous research on factors that influence PA levels [48–50]. The determinants can be categorised into pre-motivational, motivational, and post-motivational referring to factors important in determining the awareness, initiation, and maintenance phases of behaviour change, respectively [51]. The interventions, aiming to influence these factors, are computer-tailored, which means that strategies and information are adapted to the characteristics of a specific participant [52, 53] and which has been shown to be effective [54]. Individual participants thus received intervention content (multiple feedback letters) that consisted of personalised advice. For example, if a person perceives barriers for performing PA, such as pain or inconvenience to be active alone, the intervention provides persuasive information and tips especially on these aspects. Other persons receive other kinds of information, depending on their individual beliefs and characteristics.

**Table 1:** Behavioural change strategies of our included interventions [47]

Determinant	Theoretical method	Practical strategy	Intervention components	
			Print-delivered	Web-based
Awareness	Consciousness raising	Compare current PA level with similar others and PA recommendation	Graphic format wherein PA level of participants is compared with PA recommendation and PA behaviour of similar others (same age and gender, based on epidemiological evidence).	Graphic format wherein PA level of participants is compared with PA recommendation and PA behaviour of similar others (same age and gender, based on epidemiological evidence).
	Self-monitoring	Encourage monitoring of own behaviour	Logbook scheme to write down their own behaviour. An example was included in the advice. An empty form was attached to the advice.	Logbook scheme to type their own behaviour. An example was included in the advice. An empty form (pdf-format) could be down-loaded from the website.
Motivation	Active learning	Invite to formulate motivation	Space within the tailored advice to write down (intrinsically motivated) reasons to be physically active.	Space within the tailored advice to type (intrinsically motivated) reasons to be physically active.
	Social modelling	Provide role model stories about intrinsic motives to be PA	Picture of similar other (same age group and gender) with quotes about their (intrinsic) motivation to be active.	Short video of similar other (same age group and gender) who tells about their (intrinsic) motivation to be active.
Self-Efficacy	Social modelling	Provide role model stories about difficult situations and how to cope	Picture of similar other (same age group and gender) with quotes about a similar perceived difficult situation and how the role model coped.	Short video of similar other (same age group and gender) who tells about a similar perceived difficult situation and how the role model coped.
Action planning	Active learning	Invite to formulate action plan	Weekly scheme to write down plans to be PA (when, what, where, with whom). An example was included in the advice. An empty form was attached to the advice.	Weekly scheme to write down plans to be PA (when, what, where, with whom). An example was included in the advice. An empty form (PDF) could be down-loaded from the website.
Coping planning	Active learning	Invite to formulate coping plans	Scheme with space to formulate “if-then” rules. An example was included in the advice. An empty form was attached to the advice.	Scheme with space to formulate “if-then” rules. An example was included in the advice. An empty form (PDF) could be down-loaded from the website.

This article aims to answer the following research questions: 1) Do important determinants of PA, as assumed in theories underlying the design of interventions studied, appear in paths of intervention effects? 2) How do these determinants together change short- and long-term PA behaviour? 3) Do determinants occurring along paths of intervention influences, or their roles, differ between subpopulations of males and females? It is expected that previously identified important determinants, emphasised in theories underlying the design of included interventions, appear along the paths revealed by the Bayesian network model estimated in this study. However, due to the application of a modelling technique addressing a different research goal than previous analyses, new knowledge is expected to be found about the complex interaction between determinants and PA behaviour and thus about the way in which determinants mediate intervention effects. We expect that this interaction differs to some extent between males and females, resulting in different models for the subpopulations, because of previously shown moderation effects of gender [24, 36–38]. In order to verify our hypotheses, this paper presents relevant Bayesian network fragments, showing intervention influence paths on short- and long-term outcomes, learnt for the complete set of available intervention data and for male and female subpopulations.

## Methods

### Case description

This study aims to analyse the data from five different e-health intervention studies focusing on improving PA behaviour. The interventions of the included studies all have been designed based on the same theoretical methods and behaviour change strategies, as introduced in the background section (see also Fig. 1). However, as already explained, the interventions are focused on different target populations and contents are tailored based on participant characteristics. Table 2 presents the effectiveness and main characteristics of the intervention studies with respect to intervention design, which lead to differences in emphasis on facets of intervention content. Due to the complexity of tailoring where a large number of variables lead to minor content variations and intervention contents that are unique to each person, the personalisation of intervention content is beyond the scope of the current study. To get an overall overview, the current study focuses on the effects of receiving an intervention in general. The integration of corresponding datasets is based on study-overarching concept definitions. Some variables have not been measured at some time slots in particular studies and one study included does not have measurements at 12 months after the baseline, because of shorter intervention duration (see Table 2). Due to this, missing values are created in the integration, which are imputed in the analysis phase.

**Table 2:** Characteristics and effectiveness of interventions

Study index (reference)	Intervention target group	Number of participants (control group: intervention group)	Intervention delivery mode	Effectiveness at 6 and 12 months after baseline
**1**[41]	Adults aged > = 50 years	1976 (583:1385)^a^	Written	Yes. Depends on intervention content (more specifically environmental information) and differs between age-, BMI- and intention-specific subgroups
**2**[40]	Adults aged > = 50 years	2140 (411:1729)	Written and digital per individual	Yes. Depends on delivery mode and environmental conditions. Also, for some conditions moderation effects of age, gender and intention have been found
**3**[28]	Single, older adults aged > = 65	766 (0:766)	Written and digital	No. Effects at 3 months, but these have evaporated at 6 months. No measurement at 12 months
**4**[30]	Older adults aged > = 65 having chronic and/or physical limitation(s) for PA	623 (347:276)	Written and digital	Yes, although limited. Depends on degree of impairment, BMI, age and educational level
**5**[29]	Former/current cancer patients aged > = 18 years	478 (229:249)	Written and digital	Yes. Depends on cancer type and educational level, gender and age

^a^ 8 values in the intervention variable are missing and have been imputed

### Study data

**Fig. 2:**
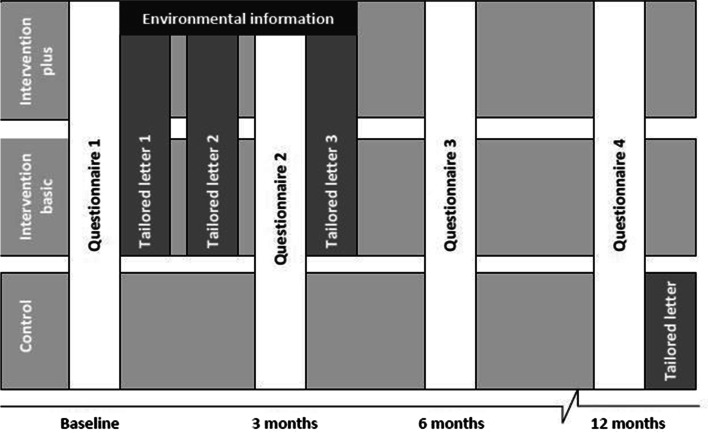
Outline of interventions including measurements [41]

Participants of the studies received questionnaires at different moments in time in order to measure baseline position and intervention effects. Apart from intervention-related information, data from questionnaires contain determinants, external factors, and measurements of PA. The measurement data were collected at the baseline (T0), which is just before participants received any tailored advice, and at 3 (T1), 6 (T2), and, in most studies, 12 (T3) months after the baseline [28–30, 40, 41], as depicted in Fig. 2. Data at T2 and T3 measure short- and long-term intervention effects, respectively. Note that the selection of data that is collected differs between the studies as well as the moments of measurement. In this paper, the analyses include an integrated dataset of a selection of the most important variables (i.e., from our theoretical perspective) derived from the measured data of the included studies. It should be noted that 4405 participants received an intervention and 1570 were part of the control group. Furthermore, participants’ age ranged from 34 to 98, but most are adults aged 50 and older (mean and median age are 65 [SD = 9.32]). During the integration of datasets, missing values are created in case a specific determinant was not measured (at a specific time point). Some variables were recoded for some studies to ensure equal meaning of values across the studies. The selection of variables includes the main determinants and demographic factors, a variable indicating the intervention condition, and PA outcomes, as described in the following subsection.

### Measurements

#### PA outcomes

To measure intervention effects on PA behaviour, the self-administered Dutch Short Questionnaire to Assess Health Enhancing PA (SQUASH) was included in the questionnaires that participants received [55], which is valid and reliable, although known for over-reporting [56]. This paper’s analyses focus on the outcome measure of total minutes of moderate- and vigorous-intensity activity per week. This variable can be derived from the raw data of the questionnaire at each point in time and Table 3 indicates in which studies it has been measured.

**Table 3:** Timeslots of measurement of determinants and outcomes (including indication of studies)

Variable/timeslot	Baseline (T0)	3 months (T1)	6 months (T2)	12 months (T3)
Self-efficacy	X (1–5)	X (1–3, 5)	X (5)	X^a^ (4)
Attitude (pros and cons)	X (1–5)	X (1–3, 5)	X (5)	X (4)
Intrinsic motivation	X (1, 2, 5)	X (1, 2)	X (5)	
Intention	X (1–5)	X (1–3)	X (1, 2, 4, 5)	X (1, 2, 4, 5)
Commitment	X (1, 2)	X (1, 2)	X (1, 2)	
Strategic planning	X (1–5)	X (1, 2)	X (1, 2, 5)	X (1, 4)
Action planning	X (1, 2, 5)	X (1, 2)	X (1, 2, 5)	
Coping planning	X (1, 2, 5)	X (1, 2)	X (1, 2, 5)	
Habit	X (1, 2, 4, 5)		X (1, 2, 4, 5)	X (1, 2, 4, 5)
Social modelling	X (1–5)	X (1–3)	X (1, 3)	X (4)
Social support	X (1–5)	X (1–3, 5)	X (3, 5)	X (4)
SQUASH outcome	X (1–5)	X (1–3, 5)	X (1–5)	X (1, 2, 4, 5)

^a^ Raw data measurements are very limited

#### Determinants

The socio-cognitive determinants of PA that were included in this paper are represented in Table 3, which also indicates for each time slot if and in which study (according to study indexes in Table 2) it has been measured. Note that in case a determinant has not been measured in a specific study at a specific time slot, this results in a missing value. This selection is based on theoretical assumptions, as presented in the introduction. Raw data were aggregated to calculate concept variables for each of these determinants to be included in this study’s analyses. It should be noted that the number of items per concept included in a questionnaire varies across the studies. Items were measured on a five-point scale, except for intention, which was measured on a 10-point scale. Item scores were recalculated, if needed, to a unipolar scale and to account for user missing values and values that are less relevant in the context of this research. The concept scales were computed as the mean of the corresponding items of that concept, with an allowed maximum of 25% missing items per scale.

#### Demographics

Furthermore, analyses include the demographics of age, gender, and educational level, which have been measured in each study included. Age is measured in years and included in analyses uncategorised, and gender is included as a Boolean variable. The educational level is categorised into low, medium, and high, according to the Dutch education system.

#### Intervention condition

We included an intervention variable that indicates whether a participant was part of the control group or the intervention group. It should be noted that the specific intervention content that participants of the intervention group receive is personalised by some additional characteristics, such as demographics and the delivery mode of the intervention, as described in the background section. However, as indicated in the case description, evaluation of personalisation of intervention content is beyond the scope of this article.

### Analysis

Our previous study on one of the intervention studies taken into account in this multiple database study, revealed that the Bayesian network model provides insight into dependency structures between the determinants of PA [33]. Therefore, to analyse pathways of correlations between factors determining PA behaviour, the temporal Bayesian network modelling technique was applied again in this paper’s analyses [57]. Bayesian networks are probabilistic models that represent relationships among variables based on conditional independences. The structure of a Bayesian network is a directed acyclic graph, where the set of nodes represents random variables. The absence of arcs between nodes represents probabilistic conditional independence among the associated variables. A causal graph can be represented by a Bayesian network [58], but it is not guaranteed that a machine-learning algorithm can find this causal graph from observational data without further causal assumptions.

The Bayesian network models learnt from the intervention data in this case study are temporal and hybrid, which means that the models have a time dimension and include discrete as well as continuous variables. In these hybrid Bayesian networks, discrete and continuous random variables are represented by a multinomial and Gaussian distribution, respectively. In the network, it is possible that continuous nodes have discrete parents, but not the other way around. Besides the characteristic of being hybrid, the Bayesian networks learnt in this case study are temporal. This means that the models are subject to the condition that arcs directed to variables in previous points in time cannot occur. We further restricted the network such that each of the demographics of age, gender, and educational level do not have parents except the other demographics. Also, the network is restricted such that the demographics are the only possible parents of the baseline characteristics except for baseline measurements themselves, and the intervention variable may have the baseline characteristics and demographics as parents only. By these restrictions that avoid obvious non-causal arcs, models are learnt whose direction of arcs can possibly be interpreted causally. However, as in any other statistical approach, we cannot rule out unmeasured confounders.

We applied a search-and-score-based algorithm that searches through candidate graphs and selects the structure that best fits the data according to a model selection criterion, in this case the Bayesian Information Criterion [59, 60]. This search process is modified to be subject to the time dimension-related restrictions described before. To handle missing data, the structural Expectation Maximisation algorithm is applied [61]. This algorithm iteratively combines structure learning with the estimation of missing values based on observed data and the model learnt in a certain iteration, and its technical performance in a PA intervention data context has been examined before [33]. To evaluate the stability of relations and their directions, we apply Efron’s Bootstrap to obtain confidence in arcs [62]. To estimate a reasonable number of bootstraps, we compared, for a chosen confidence threshold, whether the bootstrap procedure leads to a stable set of arcs measured by the structural Hamming distance. We chose the number of bootstraps where we did not observe improved stability, which ranged between 100 and 150 bootstrap samples. See Additional File 1 in the Supplementary Materials for details. To create an averaged model, we selected arcs with at least 60% confidence, which keeps the number of false negatives under control [62]. Furthermore, Friedman et al. have also shown that those arcs with high confidence are unlikely to be false positives [62].

To draw conclusions from the resulting hybrid Bayesian networks regarding determinants of PA behaviour and their inter-correlations, relevant parts of the networks are deduced. Paths between the intervention condition variable and PA short- and long-term outcome measures are distilled because the aim of this paper is to analyse how interventions affect PA behaviour and to evaluate the role of different determinants.

In order to evaluate differences among male and female subpopulations, different models of intervention effects and effects on short- and long-term PA are created. Apart from the model learnt from data of all available records, two models are learnt from data of only male and female participants, respectively. It is important to note that the general model is corrected for the factors age, education level, and gender. The subpopulation models are also corrected for age and education level, whereas the gender factor was left out of the model since it is a constant for these subsets of the data.

To analyse the resulting models, we visualise the models such that they are easy to interpret. The strength of the conditional independence relation of each arc is provided by means of asterisks in the graph. The asterisks in models presented in the result section are derived from the jack-knife bias-corrected mutual information estimates, based on complete cases, and relative by cutting off at 33 and 67% quantiles [63]. Exact values of the cut-off points are given for each model presented, and, according to these, the mutual information estimates are allocated to 3 groups. To represent the stability of relations, arc thickness varies according to the percentage of the bootstrap sample models in which the specific arc occurs. The stability of included arcs is at least 0.6 and the cut-off points to represent arcs in 4 levels of thickness are 0.7, 0.8 and 0.9. Finally, with the help of colours indicating different determinant categories, we compare findings about important determinants and paths of intervention influences from the general model to those from the subpopulation models and from the subpopulation models mutually.

## Results

This section presents the highlighted fragments of the Bayesian networks estimated based on the intervention data for both the general population learnt from all data (presented first) and the male and female subpopulations (presented second). As explained in the methods section, important parts of the Bayesian networks estimated for these cases are distilled, namely paths between the intervention condition variable and PA outcome measures. Note that the relative importance of paths is evaluated by inspecting the stability of relations indicated by arc thickness and the strength of relations indicated by the asterisks. See Additional File 2 for an overview of means and standard deviations to declare findings in the networks.

**Fig. 3:**
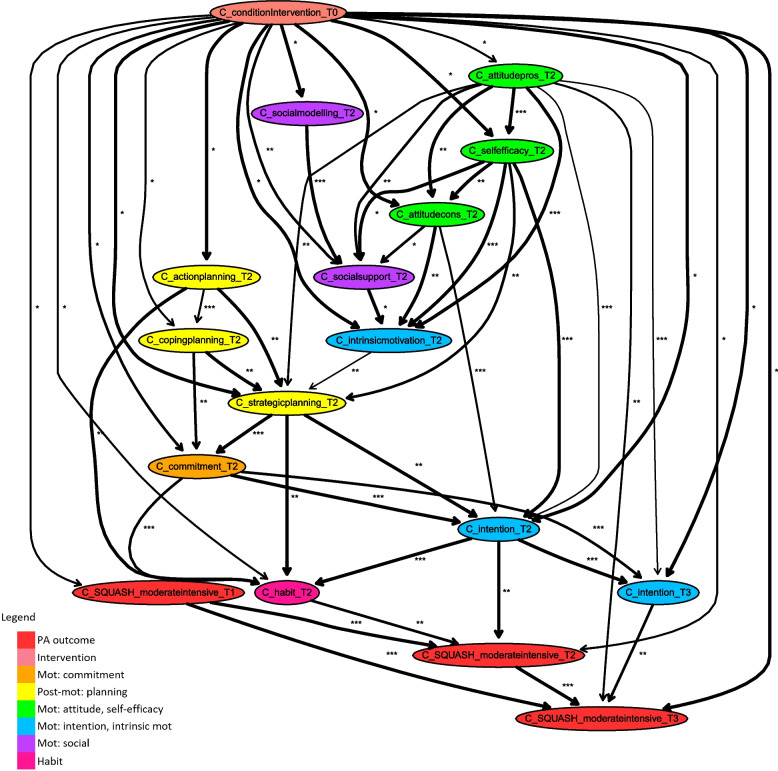
Fragment of Bayesian network consisting of all paths between intervention variable and PA outcome measures (including arcs with stability of at least 0.6)

As a starting point of our analyses, we delve into the Bayesian network model learnt for the intervention data of important socio-cognitive determinants of PA and PA outcome measures at all four time slots and corrected for the important demographic factors of age, gender and, education level. Figure 3 shows the segment of this network highlighting paths of intervention effects on PA outcome measures in the short-term and the long-term, with a stability threshold of 0.6. The mean stability of included relations is 0.91, and the median stability is 0.96. The cut-off points for bias-corrected mutual information, reported as asterisks, are 0.03 (33%) and 0.14 (67%), with minimum of 0.00 and a maximum of 0.35.

### Paths with regard to short-term PA effects (T2, 6 months)

The most important mediating determinants of intervention effects on PA outcome levels in the short-term (i.e., T2, at 6 months) are PA behaviour at T1 (i.e., 3 months) and the determinants intention and habit measured at the same point in time (T2). The intervention influences short-term PA both directly and indirectly via intention and habit, which are also strongly interrelated. Intention at T2 is influenced directly by the intervention, but also indirectly via self-efficacy, attitude (cons and pros), strategic planning, and commitment at T2. Habit at T2 is also influenced directly by the intervention but more dominantly indirectly via commitment, action planning, strategic planning, and, as mentioned before, intention at the same time point (T2). There are other paths of intervention effects on short-term PA outcomes that compose a larger chain of determinants, implying that they have a more indirect influence. These determinants influence outcomes indirectly by influencing intention and/or habit at T2 in some way. Action planning and coping planning at T2 are influenced directly by the intervention. These interrelated determinants influence strategic planning and commitment at the same point of time (T2), which both being related to (one of) the direct determinant(s) of short-term PA. Besides, intrinsic motivation at T2 appears to be a mediator of short-term intervention effects, due to its influence on strategic planning at T2. Intrinsic motivation is influenced directly by the intervention but also by the social concepts and, most dominantly, by attitude (pros and cons) and self-efficacy. Note that attitude, self-efficacy, and social support are related and that influences of social modelling in mediation paths are indirect via social support. It is remarkable that all determinants appearing in the model are influenced directly by the intervention. Also, these direct intervention effects are relatively weak, indicated by a single *, for almost all determinants, apart from that on social support. Hence, since determinants such as social support, social modelling, action planning, coping planning and, intrinsic motivation are directly influenced by the intervention and appear in longer mediation paths influencing short-term PA, they play a more indirect role.

### Paths with respect to long-term PA effects (T3, 12 months)

Similar to the short-term, the intervention directly influences long-term PA to some extent, and a large part of intervention effects on long-term PA behaviour (i.e., T3, 12 months after baseline) is determined by past behaviour. Long-term PA outcomes are also determined by other paths of intervention effects. Attitude pros at T2 is a direct mediator, and there are also longer mediation paths influencing long-term outcomes via intention at T3. Mediators along longer paths influence long-term PA via the determinant intention at the same point of measurement (T3) and/or via previous PA behaviour at T2. In turn, intention at T3 is directly influenced by the intervention and strongly determined by the previous intention level, commitment, and attitude pros at T2. Via intention (and commitment) at T2, long-term PA is influenced by paths of intervention effects that also influence short-term PA. As described before, indirect influences of the planning concepts, intrinsic motivation, attitude (pros and cons), self-efficacy, and the social concepts at T2 are revealed in this way. In paths that influence long-term behaviour via previous PA behaviour at T2, habit also occurs at T2.

### Subpopulation models compared to the general population

**Fig. 4:**
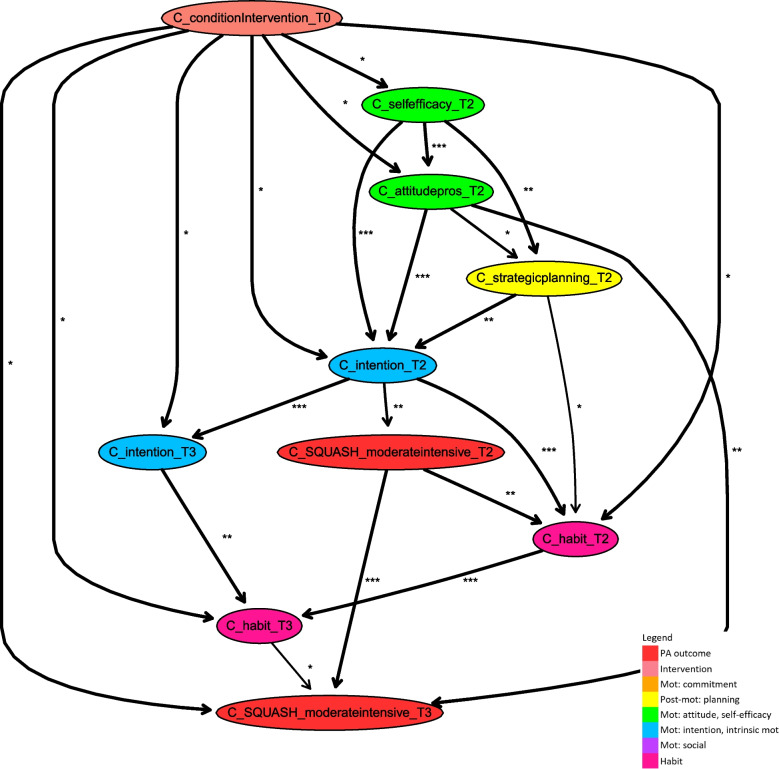
Fragment of Bayesian network learnt for the subpopulation of males, consisting of all paths between intervention and PA variables (including arcs with stability of at least 0.7)

**Fig. 5:**
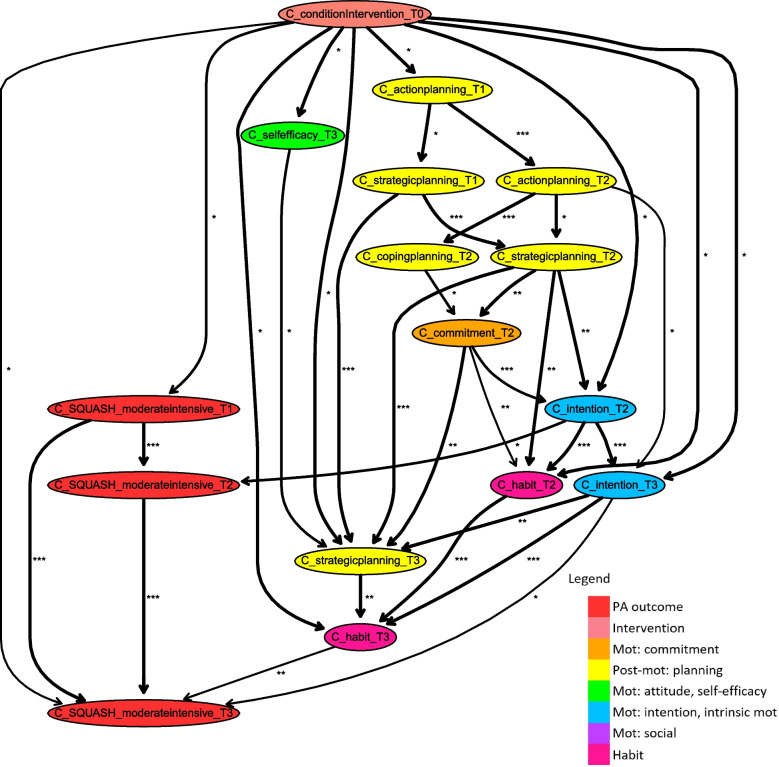
Fragment of Bayesian network learnt for the subpopulation of females, consisting of all paths between intervention and PA variable (including arcs with stability of at least 0.7)

Similarly constructed segments of the Bayesian network were learnt for data of only male and female participants with a stability threshold of 0.6. The mean stability is 0.88 and the median stability is 0.93 for males. The cut-off points for bias-corrected mutual information for males, reported as asterisks, are 0.09 (33%) and 0.16 (67%), with minimum of 0.00 and a maximum of 0.41. The mean stability is 0.91 and the median stability is 0.97 for females. The cut-off points for bias-corrected mutual information for females, reported as asterisks, are 0.10 (33%) and 0.16 (67%), with minimum of 0.00 and a maximum of 0.40. Because of the complexity of the segments, the fragments discussed here are limited to paths with a stability threshold of 0.7, which are shown in Figs. 4 and 5. The complete segments obtained with a threshold of 0.6 can be found in Additional Files 3 and 4. Because of the higher chosen stability threshold value of the discussed subpopulation models, the influences of social concepts and intrinsic motivation are not represented. More details regarding their influences can be found in the additional materials presenting subpopulation models with a lower stability threshold that is the same as the threshold in the discussed general model. Relations found in the general network turn out to unveil the averaged behaviour change mechanism across the population consisting of predominantly older adults, aged 50 or older. Looking specifically at male and female subpopulations, some parts appear to be important for a specific subpopulation and less important for the other. Especially for females, some parts of the subpopulation model are not represented in the general model. This could be because relations might be less significant in an averaged setting for the whole dataset than in a subpopulation setting for a specific part of the dataset. As a result, the model learnt for data of only males differs in some (parts of the) presented paths from that of only females. This suggests that the process of how interventions change PA behaviour is moderated by a participant’s gender to some extent. In upcoming paragraphs, we discuss the differences and similarities of short- and long-term intervention effects on PA between males and females in more detail.

### Comparison of male- and female subpopulations’ short-term behaviour

Looking at short-term PA behaviour, intention at T2 has a strong direct relation with PA outcomes at T2 and is directly influenced by the intervention for both subpopulations. For females, previous PA behaviour at T1 has a stable direct relation with PA behaviour at T2. Stronger influences on intention are via paths of other determinants. Apart from strategic planning at T2, the indirect determinants appearing along these paths differ between males and females. Next to strategic planning, for males attitude pros at T2 and self-efficacy at T2 affect intention at T2, and are directly influenced by the intervention. On the other hand, for females, commitment at T2 directly influences intention and coping planning at T2, due to intervention effects on action planning T1. Next to this, action planning at T2, strategic planning at T1, and action planning at T1 have more indirect effects. Hence, for both males and females, intention at T2 is a direct determinant of short-term PA. However, the way in which the intervention indirectly influences this determinant differs between the subpopulations; attitude and self-efficacy play a more crucial role for males, and planning seems more important for females.

### Comparison of male and female subpopulations’ long-term behaviour

For both subpopulations, the most important factor determining long-term PA at T3 is previous PA behaviour (at T2, and for females also at T1), and long-term PA is directly influenced by the intervention. Further, for the male subpopulation, attitude pros at T2 and habit at T3 directly influence long-term PA. For males, intention at T3 and habit at T2 indirectly affect long-term PA via habit at T3. These indirect determinants are influenced by the evolvement of paths of short-term intervention effects mainly via intention at T2 and directly by the intervention. For the female subpopulation, attitude does not occur in the model, and habit at T3 as well as intention at T3 are direct determinants of long-term PA. These determinants of long-term intervention effects are directly affected by the intervention and via paths evolving from short-term effects. Along these paths, habit occurs at T2 and strategic planning at T3, which are both directly influenced by the intervention. Note that strategic planning is also influenced by the intervention via self-efficacy at T3. Hence, for both subpopulations, intention and habit at T3 are determinants of long-term intervention effects on PA, although intention plays a more indirect role for males. For both subpopulations, intervention effects maintain in the long term, just as in the general model, due to influences on these determinants originating in short-term paths. An important difference between the male and female subpopulations is the appearance of strategic planning and self-efficacy in long-term paths in the female-specific model, whereas for males these determinants are only of importance in the long-term because of maintenance of their short-term effects.

## Discussion

### Main findings: determinants’ importance for PA behaviour change

This article has elaborated on the interaction between determinants that cause or mediate intervention effects on short- and long-term PA. A Bayesian network strategy was used to determine these determinant models. Referring to our first research question, the determinants of PA behaviour emphasised in the intervention’s theoretical framework do appear in paths of intervention effects in our estimated Bayesian network (see Fig. 1). More specifically, the importance of the determinants, namely attitude, self-efficacy, social influence concepts, intention, commitment, habit, intrinsic motivation, and planning concepts, is confirmed. These determinants are, even though relatively weakly, directly influenced by the intervention. This indicates that behaviour change through interventions is difficult but does occur by many different effects that initiate paths of intervention influences that, in turn, are relatively strong. Note that, although some demographic factors and baseline measurements are included in our analyses, their role is not highlighted in our research.

According to previous review studies, there was insufficient evidence for most associations between the theoretically hypothesised determinants and PA due to a lack of high-quality studies [64, 65]. Yet, at a higher level, our Bayesian network is learnt for the integrated dataset of the combination of multiple PA intervention studies. This model reveals evidence for the influence of concepts for which previous intervention research found only limited evidence, while evaluating more complex paths of behavioural change.

Besides the influences of several determinants of PA, previous PA behaviour has been confirmed to be an important factor for short- and long-term intervention effects on PA, as shown in previous research [66], and the direct influences of the intervention on PA outcomes were also seen. Although the influence of baseline PA levels falls out of the distilled fragment of the Bayesian network by definition, the importance of previous behaviour is verified by our model.

### Main findings: determinants’ role in paths of intervention effects

To answer the second research question, our Bayesian network model provides an overview of the complex interactions between these determinants and their role in improving short- and long-term PA. This confirms expectations of intervention effects on PA explained by the combination or interaction between motivational and post-motivational factors together, for which previous research has not provided indications yet [22]. Due to the evaluation of more complex paths than in previous research, new knowledge is provided with respect to the role and relative importance of these determinants in changing short- and long-term PA.

More specifically, it is observed that habit is directly correlated with short-term PA and that intention is directly correlated with both short- and long-term PA outcomes. In previous research, although intervention effects on intention have been found, mediation effects of intention on short-term PA have not been found [22]. However, the result of direct influences of intention on PA is not new for the long term. Research by van Stralen et al. [27] has shown that intention is a mediator of long-term effects on PA, confirmed by our general Bayesian network model for the complete dataset. It should be noted that the dataset of van Stralen et al. [27] is included in our multiple study dataset. The direct role of intention and habit, as shown by the Bayesian network, modifies the way in which other determinants are involved. Intention and habit are directly influenced by planning and commitment. Besides, intention is also directly influenced by the motivational determinants of attitude and self-efficacy. Social concepts and intrinsic motivation influence intention and habit via strategic planning and thus play a more indirect role. In the long term, intention is also a direct determinant of PA and is determined by the previous intention level, commitment, and attitude pros. It is shown that short-term paths evolve to long-term outcomes by influences on long-term intention and maintenance of PA levels. Therefore, earlier results on maintenance of short-term effects in the long-term are verified [37].

It turns out that motivational and post-motivational determinants that are mentioned by the theoretical framework predominantly occur in short-term paths, the effects of which evolve in influences on long-term behaviour. This nuances the theoretical assumption that pre-motivational and motivational determinants are predominantly important for short-term behaviour change while post-motivational determinants are important for long-term behaviour change [67]. Also, the described interaction between the motivational determinant intention, the post-motivational planning determinants, and PA is broader than the supposition in theories that motivational determinants affect PA via post-motivational determinants and that post-motivational determinants have a direct influence (see Fig. 1). As might be expected, the data thus suggest a more mutual and bidirectional influence between motivational and post-motivational determinants over time. Note that, besides the described differences between theoretical assumptions and our observations, the interaction between motivational determinants themselves is not even addressed in detail in the theoretical framework and turns out to be revealed in our Bayesian network model.

### Main findings: gender-specific differences

With regard to our third research question, expected differences between male and female subpopulations are confirmed. In our separate Bayesian networks estimated for these subpopulations, differences are shown with regard to the importance of specific determinants, especially with respect to the relevance of attitude for males and of planning for females. Also, the role of some determinants appearing in both models differs across males and females, such as that of self-efficacy.

To our knowledge, extensive analyses on the differences between subgroups of a general population of adults have rarely been performed earlier. Our results show important differences between males and females. This coincides with previous results on gender differences in determinants of PA, that, although limited, do show implications for developing gender-specific PA interventions [68]. Note that the theoretical framework does not include explicit assumptions regarding differences between subpopulations. Our results thus provide indications that it might indeed be good to consider adjusting these guidelines and taking into account differences among (gender-specific) subpopulations. More specifically, in our Bayesian network models, self-efficacy does play a role for females in the long term, which differs compared to its role for males in short-term paths. This is remarkable, since previous studies focusing on specific subpopulations have shown mixed results with respect to the effects of self-efficacy on PA of females, while they have shown the importance of this determinant [69, 70]. Furthermore, our models show the importance of attitude for males, while van Uffelen et al. have shown that motivating factors such as degree of competition or outdoor component of PA could be emphasised to improve the attitude of male participants [38]. For females, attitude does not seem to play a role (at a stability threshold level of 0.7), possibly because women are already highly convinced of the pros and cons of sufficient PA.

Similarities between subpopulations are found in the importance of intention and habit. The importance of habit for both males and females has been shown in earlier analyses on these subpopulations and is verified by our subpopulation models, especially due to its role in long-term paths [70]. It is remarkable, however, that habit at T3 does not appear in our general network, although our subpopulation models show its importance. The reason is the direction of the arc between PA and habit at T3 in the general network, which causes this determinant not to be selected in the distilled fragment of the Bayesian network. Also, in both subpopulation models discussed, the social concepts do not appear. The result of Van Uffelen et al.’s study, that the social aspects of PA are more important motivating factors for females than for males, was not verified, at least not given the chosen stability threshold [38].

### Methodological aspects

The current research had both strengths and issues, most of which are rather common, that need to be considered. We integrated multiple datasets to increase statistical power and to get an overall overview. Partly as a result of this integration, the combined dataset consists of a lot of missing data values. We applied an advanced algorithm for learning with missing data, the technical performance of which has been evaluated in previous research [33], and we analysed the stability of models through our extensive bootstrapping procedure.

The robust methodology we developed resulted in complex averaged Bayesian networks. To achieve our objectives, we distilled relevant fragments from the averaged models, providing an overview of significant paths of intervention effects on short- and long-term PA outcomes. Paths were included according to a chosen stability threshold value, taking false positives as well as false negatives into account. The directions of the included arcs are consistent with the bootstrap samples, where we employ temporal relationships as much as possible. This approach reduces the chance that the learning algorithm has found a Markov equivalent model of the true causal structure where some causal relationships are reversed [71]. However, among other parts of the network, there might be some relevant relations which we did not evaluate, for example because the stability of relations may depend on specific intervention conditions or demographic characteristics. Also, determinants can be highly inter-correlated, for example if because of overlap in definitions [72]. Because of that, one may find a determinant one time and another, more prominent determinant another time in a model for which less data are available. Further, directions of arcs might be switched in alternative models representing the same set of independence relations (so-called equivalence classes) and, as a result, might fall out of the distilled fragment of the Bayesian network.

### Implications for practice and future research

This paper provides new insights into the working mechanisms of PA interventions in reality compared to the theoretical design framework. It should be noted that in this research we focused on actual working mechanisms of interventions, while the extent to which people within the intervention group actually made use of the intervention is not taken into account. However, it is expected that intervention usage does not influence the structure of intervention effects, but mainly its effect size. Future research is recommended to analyse intervention usage and how this can be improved. Furthermore, we disregarded the personalisation of received intervention content and one might wonder to what extent it would be feasible to include tailoring of intervention content in analyses of effects, as each person has received a unique content. Also, our analyses are based on data from multiple studies to increase the statistical power of our analyses. Therefore, analyses are based on study-overarching definitions of constructs, although contents of included interventions are based on the same theories. Future analyses might pay attention towards the slightly different operationalisation of constructs across studies.

Based on our tentative indications, it is recommended to modify future intervention design frameworks according to new insights about the specific roles determinants play to change short- and long-term PA. Further, it is recommended to adjust interventions to be more specifically tailored to the participants’ gender to enhance intervention effects in the future. Among the less stable relations we neglected, there may be some significant ones for (other) specific subpopulations, but these relations may lose statistical power in a general model setting. The gender differences found thus need to be confirmed and declared. In order to identify subpopulations in general, it is relevant to investigate the influence of moderating factors other than gender and of a combination of factors on the structure. Other factors are for example age, educational level and the level in which people are physically impaired.

The developed analysis procedure could be refined further if there is more insight in the future regarding, for example, the performance of missing data methods in relation to methodology robustness. Besides, future analyses may need to examine network parts that we have not highlighted. This is because, for example, directions of arcs (arrows) directly related to PA outcomes might not be consistent with expectations, although arcs are included according to the direction that best fits the data. This may cause some important paths of intervention effects to be neglected in the presented Bayesian network fragments. Moreover, paths determining PA behaviour independent of intervention influences might be interesting. Extension of analyses to these cases could provide insight into important paths affecting physical behaviour that are not subject to intervention influences.

## Conclusions

To our knowledge, this is one of the first studies that unveils an overview of the structure of interactions through pathways between a PA intervention and relevant psycho-social determinants as a (Bayesian) network. The relevance of determinants mentioned in the theoretical framework is mainly confirmed in the short-term, and the study revealed the importance of determinants for which previous research has found only limited evidence, such as self-efficacy and social influence concepts. The working mechanism of interventions appears to be rather complex. Our Bayesian networks have revealed new knowledge about how determinants interact to influence short-term PA and how these effects maintain in the long term, which varies at some points compared to assumptions in theories underlying the interventions designed. In particular, the role of intention and habit in PA appears to be rather direct. Short-term habit and intention are influenced due to interactions between social influence concepts, self-efficacy, attitude, intrinsic motivation, commitment, and planning concepts. In the long term, influences are maintained mainly via effects of previous PA behaviour and by affecting long-term intention levels. Next to these general results, our gender-specific networks show how the mechanism differs for male and female subpopulations. Besides similarities of the PA behaviour change process between these subpopulations, the occurrence and role of several determinants differ. Most importantly, the role of self-efficacy differs, and attitude appears to be relevant for males, while planning concepts seem more important for females.

To conclude, our research provides new insights into the mechanism of PA behaviour change and gender-specific differences, by means of applying Bayesian networks to multiple intervention studies. These insights are valuable as they provide hypotheses of how interventions might be improved in order to enhance their effects and to improve tailoring to gender-specific subpopulations that have shown important differences in mechanisms. From a methodological perspective, this study proposes an approach for estimating and visualising stable and relevant Bayesian network fragments based on an integrated dataset with significant amounts of missing data, which could also be interesting for other application-focused research that aims to uncover complex relationships between variables.

## Supplementary Information

**Additional file 1.** Stabilisation of averaged Bayesian network models during bootstrap procedure. This graph shows the stability of averaged models for different numbers of bootstrap samples, for the general population as well as for gender-specific subpopulations.

**Additional file 2.** Overview of, in models appeared, variables’ mean (standard deviation). This table includes means and standard deviations of variables appearing in the discussed Bayesian network fragments, where the cursive ones are only present in (a) subpopulation model(s). These statistics are calculated for relevant parts of the dataset, i.e. data of participants in the control group or in the intervention group, and data of all participants or of gender-specific subpopulations.

**Additional file 3.** Bayesian network model for subpopulation consisting of males (stability threshold 0.6). This figure shows highlighted paths of the Bayesian network for the male subpopulation according to stability threshold 0.6.

**Additional file 4.** Bayesian network model for subpopulation consisting of females (stability threshold 0.6). This figure shows highlighted paths of the Bayesian network for the female subpopulation according to stability threshold 0.6.

## Data Availability

The datasets analysed during the current study are available from the corresponding author on reasonable request.

## Abbreviations

PA: Physical activity
SQUASH: Short questionnaire to assess health enhancing physical activity
