# Supplementary figures and images for: Determinants of physical activity behaviour change in (online) interventions, and gender-specific differences: a Bayesian network model
Source: Int J Behav Nutr Phys Act. 2022 Dec 19;19:155. doi: 10.1186/s12966-022-01381-2 (PMC9762063; doi:10.1186/s12966-022-01381-2)

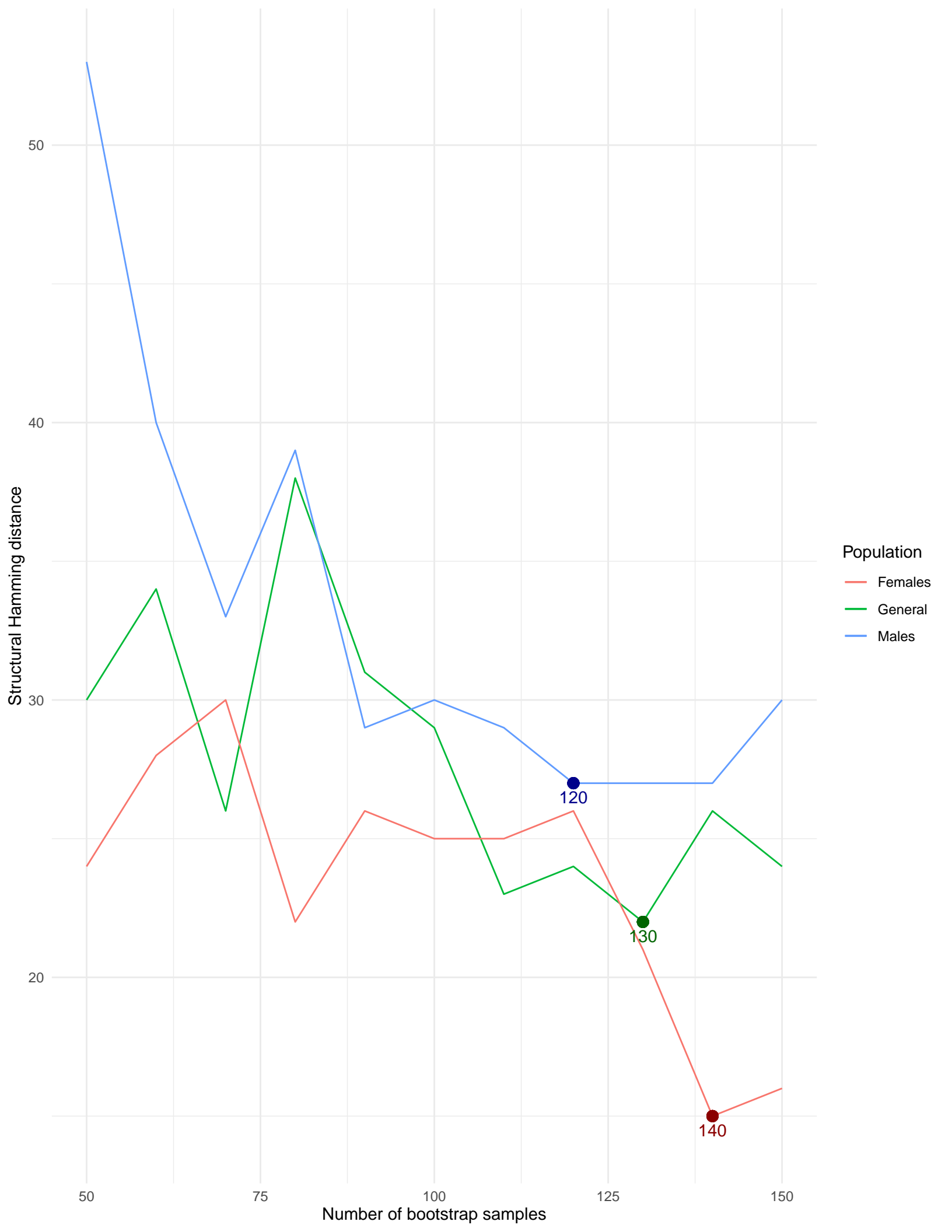

Supplement: Supplementary file 1 — Additional file 1. Stabilisation of averaged Bayesian network models during bootstrap procedure. This graph shows the stability of averaged models for different numbers of bootstrap samples, for the general population as well as for gender-specific subpopulations. [file 12966_2022_1381_MOESM1_ESM.pdf]

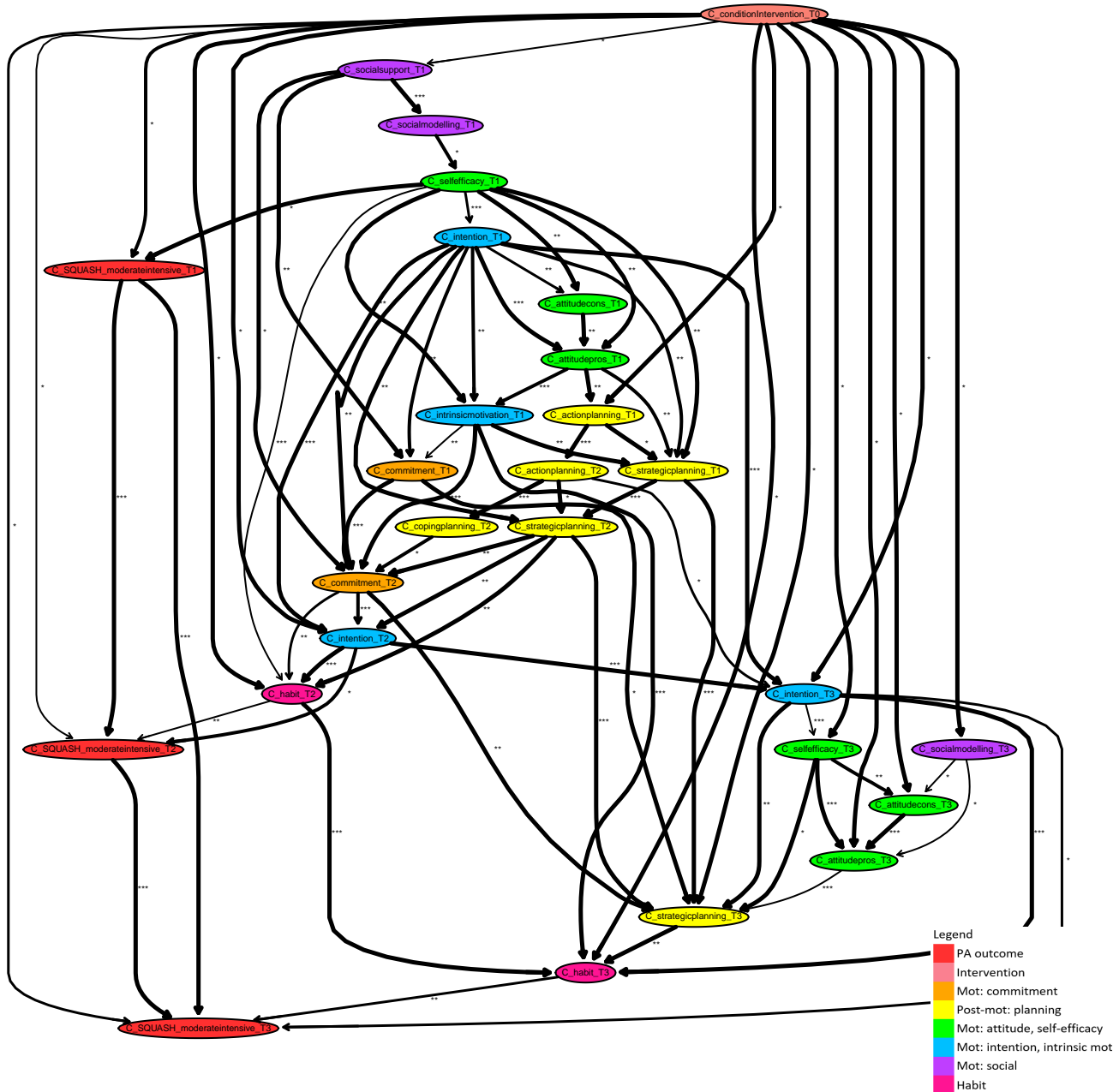

Supplement: Supplementary file 4 — Additional file 4. Bayesian network model for subpopulation consisting of females (stability threshold 0.6). This figure shows highlighted paths of the Bayesian network for the female subpopulation according to stability threshold 0.6. [file 12966_2022_1381_MOESM4_ESM.pdf]
