# Supplementary material for: Determinants of physical activity behaviour change in (online) interventions, and gender-specific differences: a Bayesian network model
Source: Int J Behav Nutr Phys Act. 2022 Dec 19;19:155. doi: 10.1186/s12966-022-01381-2 (PMC9762063; doi:10.1186/s12966-022-01381-2)
Supplement: Supplementary file 2 — Additional file 2. Overview of, in models appeared, variables’ mean (standard deviation). This table includes means and standard deviations of variables appearing in the discussed Bayesian network fragments, where the cursive ones are only present in (a) subpopulation model(s). These statistics are calculated for relevant parts of the dataset, i.e. data of participants in the control group or in the intervention group, and data of all participants or of gender-specific subpopulations. [file 12966_2022_1381_MOESM2_ESM.pdf]

Determinant (timeslot) / Subpopulation	All data		Male		Female	
	**Control**	**Intervention**	**Control**	**Intervention**	**Control**	**Intervention**
PA (T1)	777.78 (715.15)	881.58 (766.76)	872.51 (812.04)	927.01 (810.07)	664.30 (558.16)	836.89 (719.16)
PA (T2)	782.94 (734.96)	898.16 (785.57)	894.45 (835.84)	952.41 (828.26)	653.72 (571.17)	844.85 (737.70)
PA (T3)	758.27 (750.45)	827.55 (720.29)	844.83 (828.48)	893.12 (777.51)	657.34 (633.63)	758.94 (648.39)
Self-efficacy (T2)	3.80 (0.69)	3.85 (0.71)	3.82 (0.69)	3.82 (0.72)	3.64 (0.67)	4.01 (0.65)
Attitude pros (T2)	3.88 (0.56)	4.00 (0.52)	3.90 (0.57)	4.00 (0.53)	3.74 (0.45)	4.01 (0.50)
Attitude cons (T2)	3.86 (0.65)	3.98 (0.69)	3.86 (0.67)	3.99 (0.69)	3.90 (0.39)	3.92 (0.66)
Intention (T2)	7.33 (1.72)	7.66 (1.52)	7.29 (1.71)	7.58 (1.55)	7.38 (1.72)	7.74 (1.49)
Intention (T3)	7.36 (1.78)	7.66 (1.54)	7.26 (1.80)	7.63 (1.49)	7.49 (1.76)	7.68 (1.59)
Intrinsic motivation (T2)	3.75 (0.79)	3.86 (0.65)	3.76 (0.80)	3.84 (0.66)	3.62 (0.76)	3.98 (0.60)
Commitment (T2)	*3.97 (0.61)*	*4.02 (0.59)*	*3.95 (0.61)*	*3.97 (0.59)*	*3.99 (0.61)*	*4.07 (0.58)*

Strategic planning (T1)	*3.07 (0.56)*	*3.09 (0.53)*	*3.01 (0.56)*	*3.00 (0.52)*	*3.12 (0.56)*	*3.16 (0.54)*
Strategic planning (T2)	3.01 (0.55)	3.05 (0.56)	2.97 (0.55)	2.97 (0.54)	3.05 (0.55)	3.13 (0.56)
Strategic planning (T3)	*3.06 (0.57)*	*3.10 (0.53)*	*2.99 (0.55)*	*3.02 (0.50)*	*3.11 (0.57)*	*3.17 (0.54)*
Action planning (T1)	*2.84 (1.04)*	*2.87 (1.02)*	*2.89 (1.00)*	*2.74 (1.01)*	*2.80 (1.07)*	*2.98 (1.01)*
Action planning (T2)	2.82 (1.00)	2.89 (0.98)	2.77 (1.00)	2.82 (0.98)	2.88 (0.99)	2.95 (0.98)
Coping planning (T2)	2.54 (1.00)	2.56 (1.00)	2.45 (0.99)	2.45 (0.97)	2.65 (1.01)	2.68 (1.01)
Social modelling (T2)	3.53 (1.02)	3.61 (1.01)	3.52 (0.99)	3.47 (1.03)	3.54 (1.04)	3.72 (0.98)
Social support (T2)	2.24 (0.94)	2.66 (1.13)	2.26 (0.93)	2.56 (1.08)	2.03 (1.02)	2.83 (1.20)
Habit (T2)	3.41 (0.84)	3.54 (0.77)	3.34 (0.85)	3.45 (0.78)	3.48 (0.83)	3.63 (0.75)
Habit (T3)	*3.38 (0.88)*	*3.56 (0.77)*	*3.35 (0.88)*	*3.51 (0.76)*	*3.41 (0.88)*	*3.60 (0.78)*
