# Supplementary material for: Determinants of physical activity behaviour change in (online) interventions, and gender-specific differences: a Bayesian network model
Source: Int J Behav Nutr Phys Act. 2022 Dec 19;19:155. doi: 10.1186/s12966-022-01381-2 (PMC9762063; doi:10.1186/s12966-022-01381-2)
Supplement: Supplementary file 3 — Additional file 3. Bayesian network model for subpopulation consisting of males (stability threshold 0.6). This figure shows highlighted paths of the Bayesian network for the male subpopulation according to stability threshold 0.6. [file 12966_2022_1381_MOESM3_ESM.pdf]

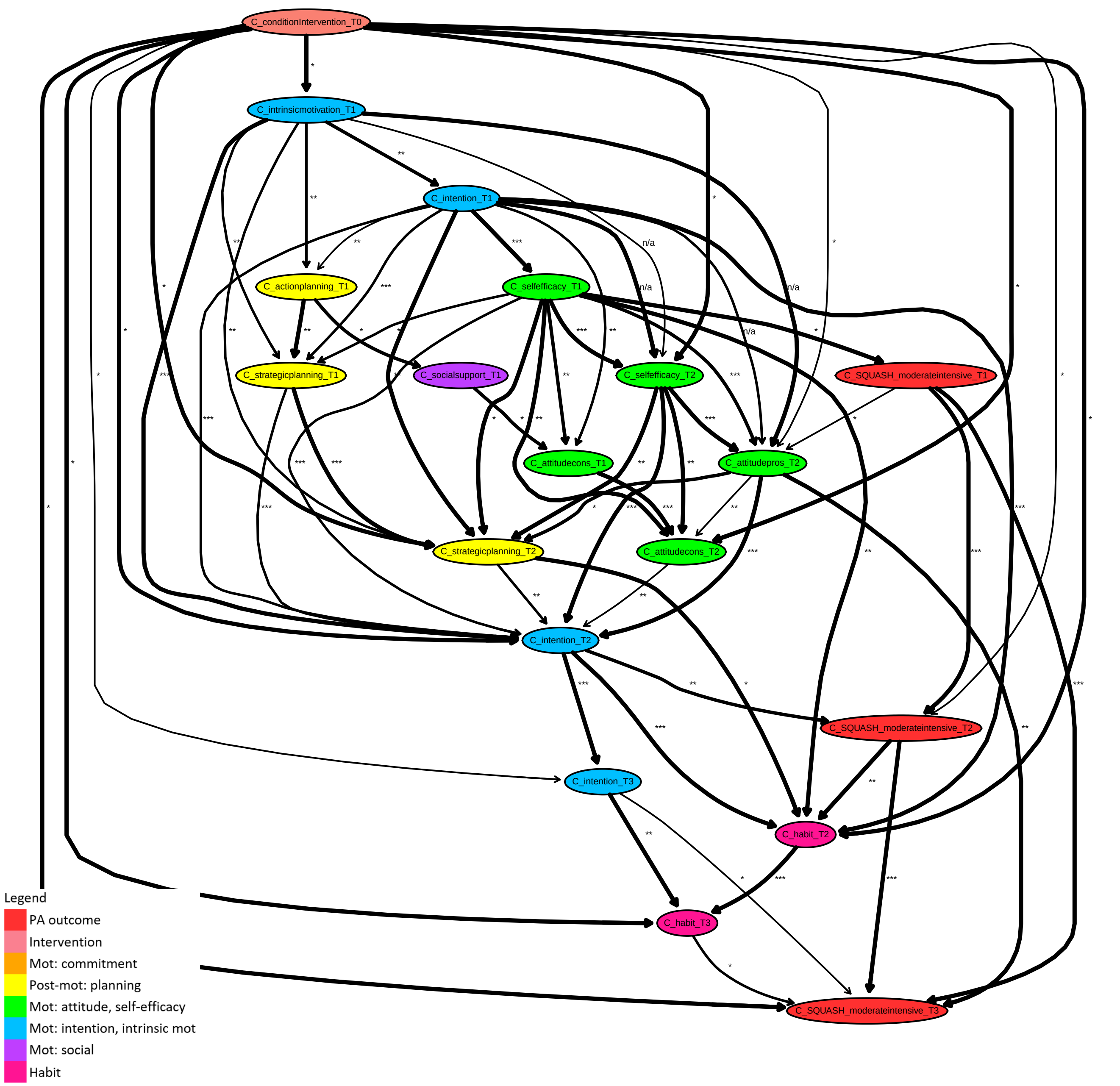

C_conditionIntervention_T0
C_intrinsicmotivation_T1
C_intention_T1
C_actionplanning_T1
C_selfefficacy_T1
C_strategicplanning_T1
C_socialsupport_T1
C_selfefficacy_T2
C_SQUASH_moderateintensive_T1
C_attitudecons_T1
C_attitudepros_T2
C_strategicplanning_T2
C_attitudecons_T2
C_intention_T2
C_SQUASH_moderateintensive_T2
C_intention_T3
C_habit_T2
C_habit_T3
C_SQUASH_moderateintensive_T3
n/a
Legend
PA outcome
Intervention
Mot: commitment
Post-mot: planning
Mot: attitude, self-efficacy
Mot: intention, intrinsic mot
Mot: social
Habit
